# Supplementary material for: Amino Acid Nanofibers Improve Glycemia and Confer Cognitive Therapeutic Efficacy to Bound Insulin
Source: Pharmaceutics. 2021 Dec 29;14(1):81. doi: 10.3390/pharmaceutics14010081 (PMC8778970; doi:10.3390/pharmaceutics14010081)
Supplement: Supplementary file 1 [file pharmaceutics-14-00081-s001.zip › pharmaceutics-1498826 Supplementary Material/pharmaceutics-1498826 supplementary - updated.pdf]

pharmaceutics

MDPI

Supplementary Materials: Amino Acid Nanofibers Improve Glycemia and Confer Cognitive Therapeutic Efficacy to Bound Insulin

Aejin Lee, McKensie L. Mason, Tao Lin, Shashi Bhushan Kumar, Devan Kowdley, Jacob H. Leung, Danah Muhanna, Yuan Sun, M Joana Ortega-Anaya, Lianbo Yu, Julie Fitzgerald, A Courtney DeVries, Randy J. Nelson, Zachary M Weil, Rafael Jiménez-Flores, Jon R. Parquette and Ouliana Ziouzenkova

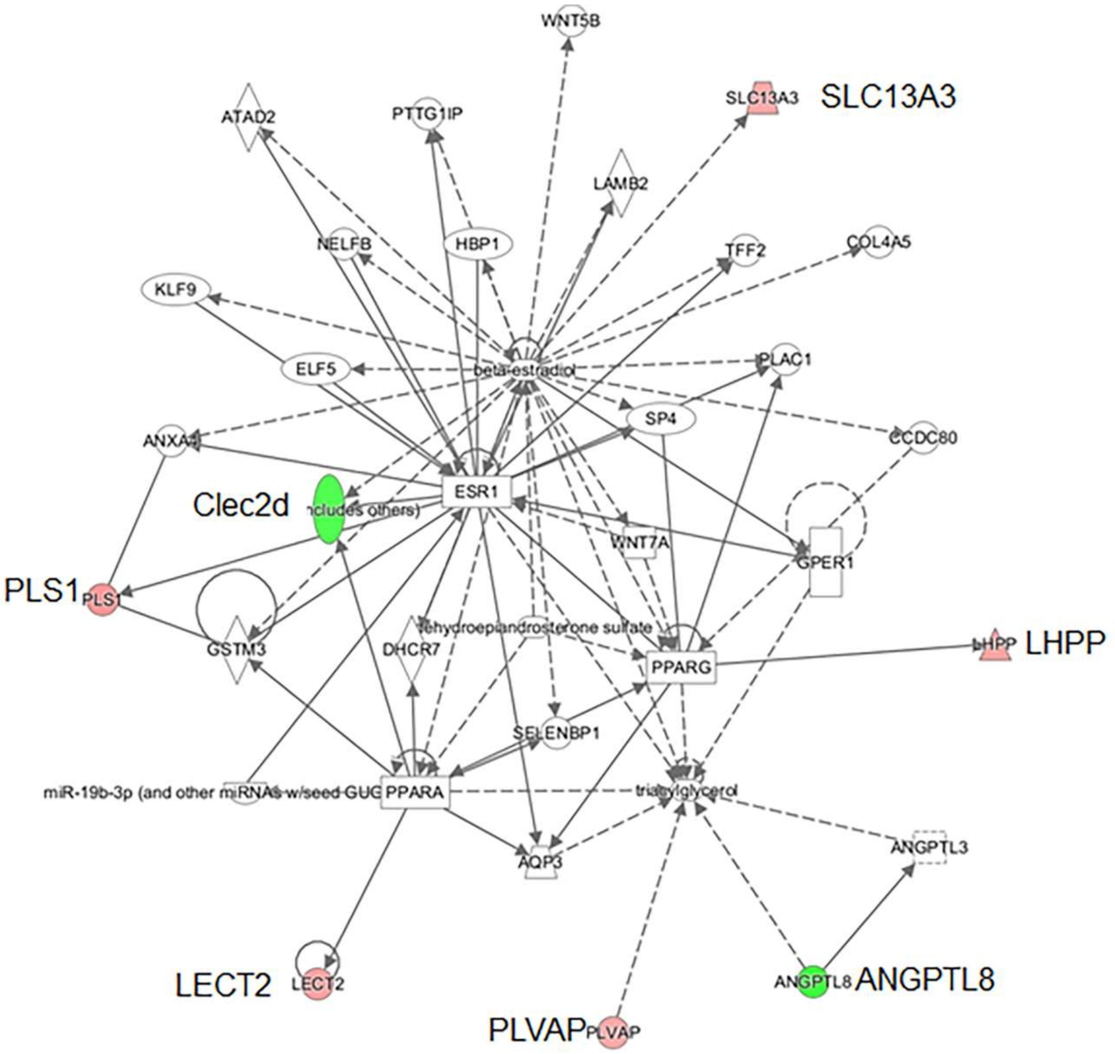

Figure S1. AAC2-hINS treatment regulated distinct pathways compared to free AAC2. Ingenuity pathway analysis (IPA) was performed based on the statistically different genes predicted metabolic hubs that were distinctly regulated in response to free AAC2 vs. AAC2-hINS treatments. Green shapes indicate higher expression in AAC2-hINS vs. AAC2 treatment groups, such as *Sleg2d* and *Angptl8*. Red shapes show the higher expression of genes in mice treated with free AAC2 vs. AAC2-hINS, such as *Slc13a3*, *Pls1*, *Lect2*, *Lhpp*, and *Plvap*.

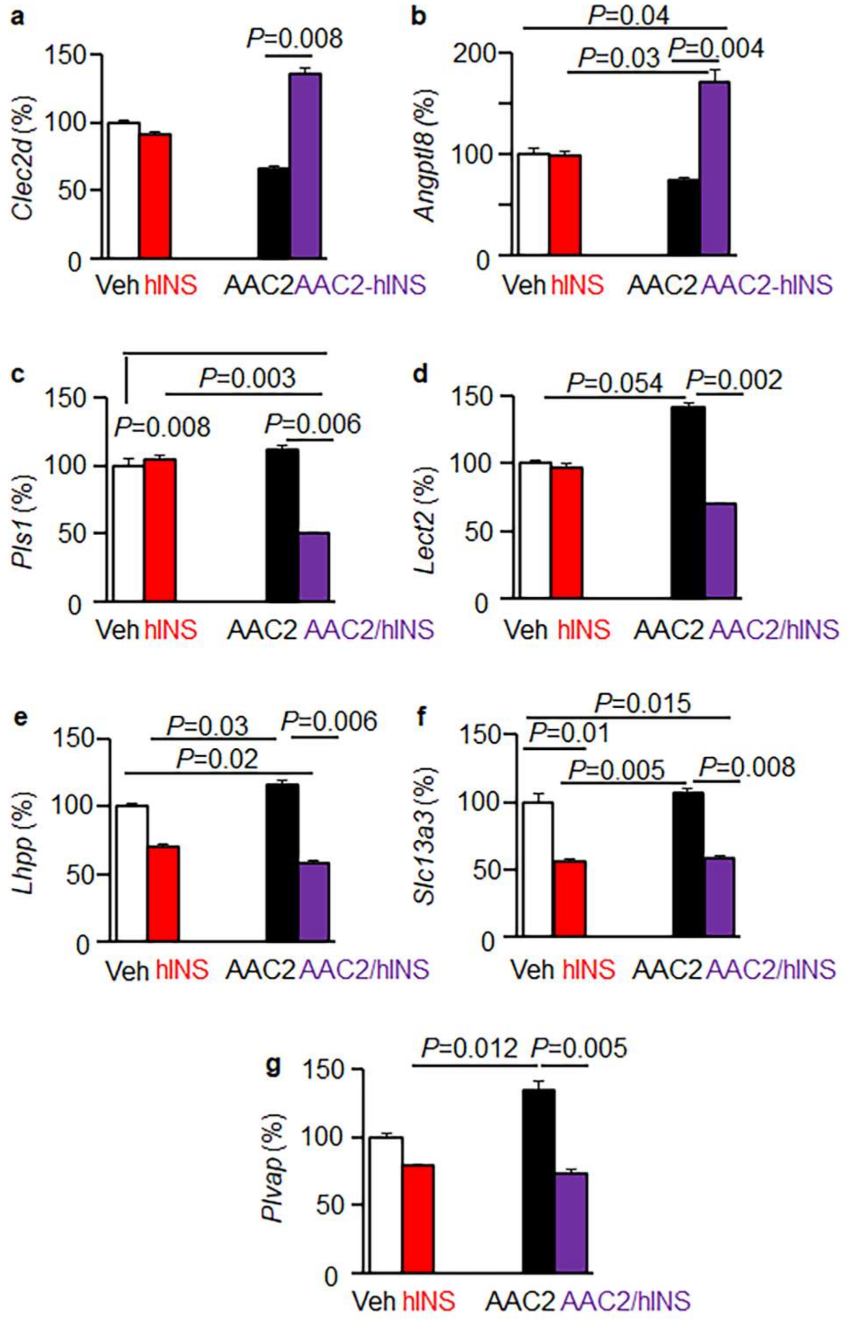

Figure S2. Expression of genes underlying specific responses in free AAC2 vs AAC2-hINS-treated mice according to IPA analysis. Comparison of the expression of hepatic genes, that were significantly different from control (Veh, not shown, $n = 3$, cut off were ≥ 1.5 folds difference and $p < 0.01$) across STZ mice treated with AAC2 (black circle), hINS (red circle), and AAC2-hINS (purple circle), ($n = 3$/group). Gene expression was analyzed by Affymetrix GeneChip followed by Ingenuity pathway analysis. (**a**,**b**) *Slec2d* and *Angptl8*,- the group of genes that were significantly induced only in in mice treated with AAC2-hINS complex compared to other groups. (**c**,**d**) *Pls1* and *Lect2*,- the group of genes that were significantly reduced only in mice treated with AAC2-hINS complex mice. (**e**,**f**,**g**) *Lhpp*, *Slc13a3*, and *Plvap* gene expression was similar between free hINS- and AAC2-hINS treated mice but were reduced compared to a control and AAC2-treated groups.

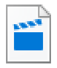

Free INS 41d post tr.mp4

Control 41 post tretment before eut.r

AAC2 hINS complex Videos for Suppleme

AAC2 41d post tr NO INS added.mp4

Video

Mice treated with AAC2-hINS display improved morphological and behavioral characteristics compared to mice treated with free hINS or AAC2 and dying control mice with STZ-induced diabetes. Mice were treated with STZ as described in Schematics in Figure 4a. Four videos showed the cages of mice treated with free AAC2, free hINS, AAC2-hINS, or remain an untreated control. Two last mice in STZ control non treated group lost more than 20% weight, displayed distress, and reached the mortal stage of T1D. STZ-mice treated with free hINS and AAC2 survived, were active but their hinged posture suggest a distress. STZ-mice treated with AAC2-hINS showed normal growth, activity, and behavior.
